# Burnt sugarcane harvesting is associated with rhinitis symptoms and inflammatory markers^☆^

**DOI:** 10.1016/j.bjorl.2018.02.008

**Published:** 2018-04-05

**Authors:** Iara Buriola Trevisan, Ubiratan de Paula Santos, Marceli Rocha Leite, Aline Duarte Ferreira, Bruna Spolador de Alencar Silva, Ana Paula Coelho Figueira Freire, Gabriel Faustino Santa Brigida, Ercy Mara Cipulo Ramos, Dionei Ramos

**Affiliations:** a Universidade Estadual Paulista (Unesp), Faculdade de Ciências e Tecnologia, Departamento de Fisioterapia, Presidente Prudente, SP, Brazil; b Universidade de São Paulo (USP), Faculdade de Medicina, Instituto do Coração (InCor), Divisão Pulmonar, São Paulo, SP, Brazil

**Keywords:** Biomass, Occupational exposure, Particulate matter, Rhinitis, Inflammation mediators, Biomassa, Exposição ocupacional, Material particulado, Rinite, Mediadores da inflamação

## Abstract

**Introduction:**

Burnt sugarcane harvesting requires intense physical exertion in an environment of high temperature and exposure to particulate matter.

**Objective:**

To evaluate the effects of burnt sugarcane harvesting on rhinitis symptoms and inflammatory markers in sugarcane workers.

**Methods:**

A total of 32 male sugarcane workers were evaluated with questionnaire for rhinitis symptoms, and for inflammatory markers on peripheral blood and nasal lavage, in the non-harvesting, and 3 and 6 months into the sugarcane harvesting period. Weather data and particulate matter fine concentrations were measured in the same day.

**Results:**

The particulate matter concentrations in sugarcane harvesting were 27 (23–33 μg/m^3^), 112 (96–122 μg/m^3^), and 63 (17–263 μg/m^3^); 24 h temperatures were 32.6 (25.4–37.4 °C), 32.3 (26.7–36.7 °C) and 29.7 (24.1–34.0 °C) and relative humidities were 45.4 (35.0–59.7%), 47.9 (39.1–63.0%), and 59.9 (34.7–63.2%) in the non-harvesting period, three and 6 months of the harvesting period. The age was 37.4 ± 10.9 years. The prevalence of rhinitis symptoms was significantly higher at 3 months of the harvesting period (53.4%), compared to non-harvesting period (26.7%; *p* = 0.039) and at 6 months into the harvesting period (20%; *p* = 0.006). Concentrations of interleukin 6 (IL-6) in nasal lavage increased after 3 months of the harvesting period compared to the non-harvesting period (*p* = 0.012). The presence of rhinitis symptoms, after 3 months of the harvesting period, was directly associated with blood eosinophils and inversely associated with neutrophils.

**Conclusions:**

After 3 months of work in burnt sugarcane harvesting the prevalence of rhinitis symptoms and IL-6 in nasal lavage increased. Furthermore, eosinophil counts were directly associated with the rhinitis symptoms in the period of higher concentration of particulate matter.

## Resumo

**Introdução:**

A colheita de cana-de-açúcar queimada requer esforço físico intenso em um ambiente com altas temperaturas e exposição a material particulado.

**Objetivo:**

Avaliar os efeitos da colheita de cana-de-açúcar queimada nos sintomas de rinite e marcadores inflamatórios de cortadores de cana-de-açúcar.

**Método:**

Foram avaliados 32 cortadores de cana-de-açúcar do sexo masculino por meio de um questionário para sintomas de rinite, e marcadores inflamatórios em sangue periférico e lavado nasal,no período de entressafra, e em 3 e 6 meses após o início da colheita da cana-de-açúcar. Os dados climáticos e as concentrações de material particulado fino foram medidos no mesmo dia.

**Resultados:**

O material particulado fino na entressafra e em 3 e 6 meses de safra foi 27 (23–33 μg/m^3^), 112 (96–122 μg/m^3^) e 63 (17–263 μg/m^3^), respectivamente; a temperatura de 24 horas foi 32,6 (25,4°-37,4 °C), 32,3 (26,7°-36,7 °C) e 29,7 (24,1°-340 °C) e a umidade relativa do ar foi 45,4 (35,0%-59,7%), 47,9 (39,1%-63,0%), e 59,9 (34,7%-63,2%), na entressafra, 3 e 6 meses após o início da colheita. A idade foi de 37,4 ± 10,9 anos. A prevalência de sintomas de rinite foi significativamente maior em 3 meses da S (53,4%), comparado com a entressafra (26,7%; *p* = 0,039) e 6 meses da safra (20%; *p* = 0,006). As concentrações de interleucina 6 (IL-6) no lavado nasal aumentaram após 3 meses do início da colheita comparado com a entressafra (*p* = 0,012). A presença de sintomas de rinite, após 3 meses do início da colheita, foi diretamente associada com eosinófilos e inversamente associada com neutrófilos.

**Conclusões:**

Após 3 meses do início da colheita da cana-de-açúcar queimada, houve aumento na prevalência de sintomas de rinite e IL-6 em LN. Além disso, as contagens de eosinófilos foram diretamente associadas aos sintomas de rinite no período de maior concentração de material particulado.

## Introduction

Brazil is the largest producer of sugar and ethanol from the cultivation of sugarcane.^1^ More than 50% of cultivation is concentrated in the south-central region of the country,^2^ with a production of 571.34 million tons of sugarcane in the 2014/2015 harvest.^3^

Despite technological advances with mechanized harvesting in the State of São Paulo, sugarcane is still harvested manually in several states of Brazil.^4^ To facilitate manual cutting and eliminate the incidence of accidents by poisonous animals nocturnal burning of the sugarcane straw is carried out in the harvest period.^5^, ^6^ This practice releases into the environment a considerable percentage of gaseous and particulate matter (PM) which is consequently inhaled by the rural worker.^7^, ^8^, ^9^, ^10^, ^11^

The work of manual cutting of sugarcane requires an intense physical exertion of the worker. Furthermore, according to the Brazilian Regulatory Standards 31^12^ the use of facial protection masks is not standard. Thus, some workers use cloths on their faces to avoid respiratory discomfort, however there is no scientific evidence that these strategies prevent inhalation of pollutants from burning. In addition harvesting is performed under high climatic temperature and low humidity, which causes adverse effects such as physical and psychological stress, and an increase of the respiratory effort.^8^, ^9^, ^10^, ^11^

The first line of defense of the respiratory system is the nose, which is responsible for humidification, heating and filtration of circulating air, and thus is vulnerable to the effects of air pollution. When individuals are exposed to an environment with the presence of pollutants, it increases the risk of developing rhinitis symptoms, either by irritant factors or an immune-mediated mechanism.^13^, ^14^, ^15^

Rhinitis is defined as the presence of nasal congestion, rhinorrhea, sneezing and/or nasal itching.^15^, ^16^ The pathogenesis of rhinitis is not well understood, but studies show an increase of inflammatory cells such as eosinophil, neutrophils, lymphocytes and increased interleukins proinflammatory in the nasal mucosa.^17^, ^18^, ^19^, ^20^ Among the interleukins proinflammatory, the interleukin 6 (IL-6), after its exposure to PM, increases in the acute phase of the inflammation, neutralizing pathogens and minimizing tissue damage.^21^ IL-4, on the other hand, has anti-inflammatory action, blocking the synthesis of several interleukins, including IL-6, with the aim of producing antibodies and immune reactions against allergens.^22^ Both are present in work-related rhinitis caused by allergic or non-allergic (irritant) factors present in the workplace.^16^

Despite the evidence on the deleterious effects of work on the cutting of burnt sugarcane, studies involving upper airway disorders are scarce.^23^, ^24^, ^25^, ^26^ Therefore the evaluations performed on the third and the sixth month after the beginning of the harvest aimed to identify whether the exposure time to fine particulate matter (PM_2.5_) and climatic variations during the harvesting may be responsible for the increase of rhinitis symptoms and inflammatory markers and whether these changes persist until the end of the harvest, contributing to the implementation of possible prevention measures in this population. Thus, the objective of the present study was to evaluate the effects of burnt sugarcane harvesting on rhinitis symptoms and inflammatory markers in sugarcane workers.

## Methods

**Figure 1:**
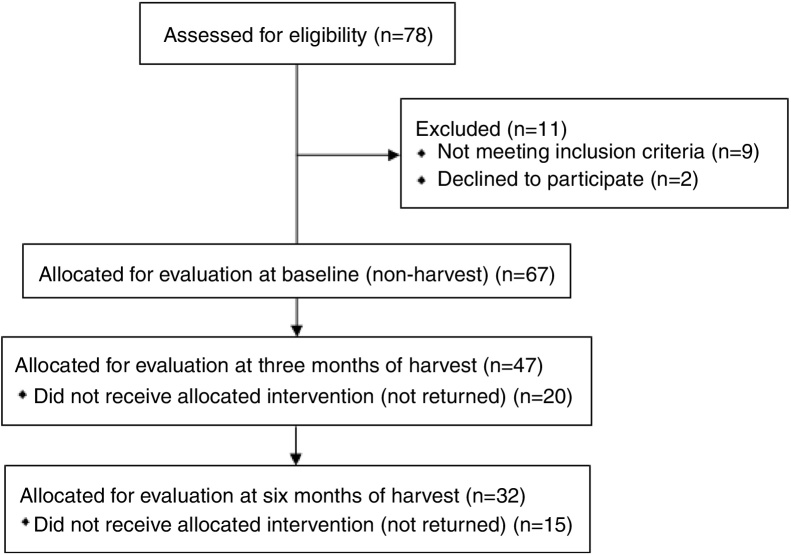
Flow chart of the study.

A longitudinal study with repeated measures was performed with sugarcane workers from a sugar and alcohol plant. Initially 78 interested sugarcane workers were eligible for evaluation. Out of these, 67 individuals were included at baseline (non-harvesting), however 32 individuals completed all assessments (Fig. 1).

The exclusion criteria were pre-existing chronic lung diseases, upper airway infection at time of evaluation, and regular use of anti-inflammatory drugs.

All individuals were previously informed about the objectives and procedures of the study and, after an agreement and a signature of the free and informed consent according to the Helsinki Declaration, they participated voluntarily and effectively in the study. All procedures were approved by the Committee for Ethics in Research (opinion n° 644.598).

All procedures were performed in the field at 7:00 am in three periods: Non-harvesting (NHP – sugarcane plantation, when there is no burnt sugarcane harvesting, March–April/2014), and Harvesting (HP – 3 and 6 months after the beginning of the harvest, when there is manual cutting of burnt sugarcane, July–October/2014). This process of burnt sugarcane occurs at night in the HP period and its duration depends on the extent of the planted area between the months of May to November. Harvesting occurs on the following day of its burning, only in this period. However the sugarcane workers also performed the planting in the NHP period (January to April), thus they work in both periods during the year.

Volunteers were previously interviewed through questionnaires, extracting general data (personal data, smoking history) and occupational activity (working time frame, amount of sugarcane harvested/day and working time in the sector).

During the three periods, the volunteers were evaluated for the presence of rhinitis symptoms through a questionnaire with four questions about nasal symptoms (nasal itching, sneezing, nasal congestion and rhinorrhea). When two or more symptoms were reported, the individual received a diagnosis of rhinitis by a medical specialist. For the analysis of inflammatory markers (IL-6 and IL-4), a collection of Nasal Lavage (NL),^26^ was obtained and it was analyzed by the method enzyme linked immune sorbent assay (ELISA, eBioscience, Affymetrix, California, United States), using Ready-SET-Go kit for the analysis of IL-6 and High Sensitivity kit for the IL-4, according to the manufacturer's instructions. For the total count of eosinophil and neutrophils in venous blood samples, the automated counting by XT-1800 equipment, Sismex, model Ap.XT17476, Japan, was analyzed from the collection of four milliliters of blood from the antecubital vein into a vacuum tube.

The monitoring of PM_2.5_ was performed during the working day (7:00 am to 3:00 pm) of sugarcane workers in two to three days, in each specified period. The analysis was measured with a mass spectrophotometer DUST TRAK Aerosol Monitor, Modelo 8533 (TSI Inc., Mn. EUA), calibrated daily, with a flow calibration of 1.7 L/min generating a PM value expressed in μg/m^3^.^24^

The temperature and relative humidity data were recorded simultaneously to those of PM_2.5_, through a thermo-hygrometer DataLogger model DHT-2261 Full Range. This device collects temperature and relative humidity data minute by minute. The equipment was positioned at a height of approximately one meter and a distance of 2–3 m from the workers, therefore not interfering in the work.

Data are expressed as mean, standard deviation (SD) when normally distributed or at median and interquartile range (difference between the 1st and 3rd quartiles, IQR), except for the categorical data that was expressed in absolute values and percentage. For comparisons between the prevalence of rhinitis symptoms in the periods the Mc Nemar test was performed. The results of continuous data obtained during the NHP and after three and six months of HP were compared by repeated measures with post hoc Bonferroni (sphericity assumed or Greenhouse–Geisser test by the Mauchly test). The Friedman test with post hoc Dunn was performed when there was a violation of the sphericity of data. Analysis generalized linear models (Generalized Estimating Equations – GEE) for dependent data with adjustments for age, body mass index (BMI) and smoking, using as reference the NHP was performed to identify possible relationships between rhinitis symptoms and inflammatory markers. Statistical analyses were carried out using the SPSS statistical package, version 15 (SPSSInc., Chicago, IL, USA) and GEE models were carried out using R program. *p*-value less than 0.05 was considered statistically significant for all tests.

## Results

Sixty-seven male sugarcane workers were evaluated in the NHP, at 3 and 6 months after the beginning of HP. The mean age was 37.4 ± 10.9 years. Among the volunteers, 52.2% (*n* = 35) never smoked, 23.9% (*n* = 16) were light to moderate smokers, smoking on average 9.3 ± 7.0 cigarettes/day (8.5 ± 8.7 packet-years) and 23.9% (*n* = 16) reported to be ex-smokers (9.5 ± 11.4 pack-years) (Table 1).

**Table 1:** General characteristics of sugarcane workers in the NHP (*n* = 67).

*Demographic and anthropometric characteristics*	
Age (years)	37.4 ± 10.9
Weight (kg)	72.0 ± 11.2
Height (cm)	169.2 ± 7.0
BMI (kg/m^2^)	25.2 ± 3.9

*Occupational activity*	
Years worked	11.2 ± 7.9
Sugarcane harvested/day (tons)	9.4 ± 2.6

Data expressed in mean (SD); IMC, Body Mass Index (BMI).

**Figure 2:**
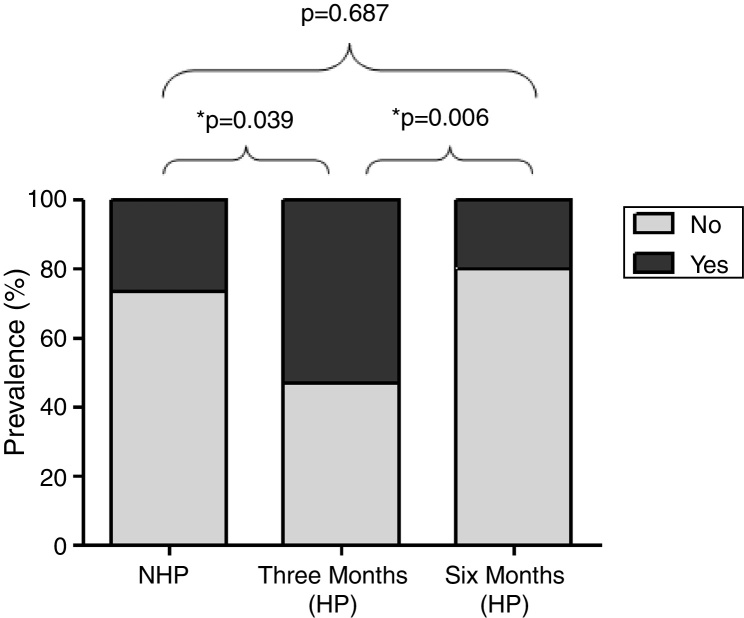
Prevalence of rhinitis symptoms in 32 sugarcane workers during NHP, three and six months of HP. McNemar's test (*p < 0.05).

Fig. 2 shows the prevalence of rhinitis symptoms in 32 participants evaluated in the three periods. It was significantly higher 3 months after the beginning HP (53.4%) compared to the NHP (26.7%, *p* = 0.039) and 6 months after the beginning HP (20%, *p* = 0.0006).

Table 2 data of inflammatory markers in the NL, eosinophil and neutrophils circulating are described. It was observed that there was a significant increase in the concentrations of IL-6 in LN after 3 months of HP, compared to NHP (*p* = 0.012). The eosinophil and neutrophil counts did not present significant differences between the periods.

**Table 2:** Analysis of repeated measures in the nasal and systemic inflammatory profile in 32 sugarcane workers during the NHP and 3 and 6 months of HP.

	NHP	3 months of HP	6 months of HP	*p*-value
*Nasal inflammation*				
IL-6 (pg/mL)	2.52 (1.77–4.09)	4.21 (2.82–5.98)^a^	3.14 (2.58–6.18)	0.046^b^
IL-4 (pg/mL)	2.50 (1.42–5.55)	2.43 (1.56–5.37)	3.29 (1.35–8.21)	0.688

*Systemic inflammation*				
Eosinophil (mm^3^)	158 (84.08–289.50)	183.90 (122.73–397.23)	205.70 (152.48–348.33)	0.427
Neutrophil (mm^3^)	2936.05 (2254.15–3655.35)	3289.55 (2310.93–4012.63)	2745.50 (2336.03–4017.53)	0.793

Data expressed in median (IQR, 1st and 3rd quartiles).

a Three months of HP difference of NHP (*p* = 0.012).

b Repeated measures with post hoc Bonferroni (*p* < 0.05).

Table 3 shows the multiple regression data, demonstrating that in the HP (after 3 months) the presence of rhinitis symptoms was directly associated with blood eosinophil and inversely associated with neutrophils in the analysis by GEE.

**Table 3:** Estimated effects of HP, neutrophils and eosinophils in the rhinitis symptoms (*n* = 32).

	Variable effect^a^	*β*	SE	*p*-value^b^
Rhinitis symptoms	Three months (HP)	1.2541385314	1.307246	0.0056
	Neutrophils	−0.4122999	0.167252	0.00568
	Eoinophils	1.495592	0.611645	0.0141

*β*, regression coefficient; SE, standard error.

a NHP Reference; Generalized Estimating Equations (GEE), adjusted for age, Body Mass Index (BMI) and smoking.

b *p* < 0.05.

The concentrations of PM_2.5_ were 27.0 (23.0–33.0 μg/m^3^), 111.5 (96.5–122.3 μg/m^3^) and 63.2 (17.1–262.6 μg/m^3^), in NHP, 3 and 6 months of HP, respectively (*p* < 0.0001). The measures of the mean temperature within 24 h were 32.6 (25.4–37.4 °C), 32.3 (26.7–36.7 °C) and 29.7 (24.1–34.0 °C) and relative humidity within 24 h were 45.4 (35.0–59.7%), 47.9 (39.1–63.0%), and 59.9 (34.7–63.2%) in NHP, on the third and sixth months of HP, respectively (*p* < 0.0001).

## Discussion

The data from this study revealed that the period of three months of the harvest is characterized with the highest concentrations of PM_2.5_ registered in the field of sugarcane and low average ambient temperature in 24 h, therefore sugarcane workers have a higher prevalence of rhinitis symptoms and increase in IL-6 concentrations in NL. In addition, rhinitis symptoms were directly associated with eosinophil and negatively with neutrophils.

Presence of the rhinitis symptoms can be influenced by endogenous factors (e.g. genetic factors, immune deficiencies, mucociliary clearance dysfunction) as well as age and gender.^27^ However, exogenous triggers (e.g. ex., ambient air pollution, viruses and bacteria, temperature, relative humidity, and smoking), especially in the workplace have been a research subject in the development of rhinitis.^28^, ^29^, ^30^

The effects on respiratory health of sugarcane workers have been described in the literature,^9^, ^11^, ^23^, ^24^, ^25^, ^31^ and probably can be attributed mainly to the inhalation of PM resulting from the sugarcane burning and the re-suspension of soil particles by the movement and activity of workers and by the movement of trucks. However, no study has isolated the effects of pollutants, temperature and physical effort, factors that may contribute to influence the findings.

Studies have shown that the frequency of symptoms related the rhinitis may be influenced by the dose response of the exposure to which individuals are subjected, changing according to the types of pollutants and their conditions of use, besides confounding factors such as exposure to cigarette smoke.^32^ Thus, changes in the concentration of pollutants released by the sugarcane burning can influence the reports of respiratory symptoms.^8^, ^23^, ^33^, ^34^ This may explain the higher prevalence of rhinitis symptoms in the evaluation after 3 months of HP compared to NHP and 6 months of HP, which explains what level of exposure was able to trigger such changes besides the atopy.

The study by Prado et al.^23^ carried out with sugarcane workers and healthy volunteers of a city neighboring the plantation field observed that sugarcane workers presented an increase on symptoms such as wheezing, wheezing with breathlessness, waking with cough and nasal allergies and high fever during the harvesting period compared to non-harvesting.

The study by Gascon et al.^34^ evaluated 104 workers at one refinery of sugarcane exposed to dust, bagasse and their bio contaminants, where they observed that during the harvesting period there was an increase in reports of wheezing, shortness of breath, ocular problems and rhinitis among those exposed to bagasse and dust. Another study carried out with children exposed to biomass burning observed a prevalence of 11% for asthma and 33.2% for rhinitis symptoms, which was more frequent in the months between June and October, a period coinciding with the sugarcane burning.^35^

One of the possible mechanisms involved in increasing the incidence of rhinitis may involve exposure to PM, which is increased during work that requires greater physical effort, in addition to the sugarcane burning. Studies suggest that inhalation may cause damage to different levels of the respiratory system, causing inflammatory airway diseases, including allergic rhinitis or chronic cough.^36^, ^37^, ^38^ The study of Hong et al.,^39^ which evaluated the toxic effect of atmospheric PM_2.5_ on human nasal epithelial cells, observed changes in morphology and decreased cell viability of nasal epithelial cells after exposure. In addition, it increased levels of reactive oxygen species, reduced intracellular antioxidant enzyme activity and induced the expression of inflammatory cytokines IL-13, IL-6, IL-8, TNF-α. These results revealed that PM_2.5_ induced oxidative stress and inflammatory response, which contributes to nasal epithelial barrier dysfunction.

The air pollution factor in the present study may have been a decisive factor to IL-6 to cause an increase in nasal lavage. The study of Steenhof et al.^40^ carried out with 31 volunteers at five sites (two traffic sites, an underground train station, a farm and an urban background site) was aimed to assess nasal and blood pro-inflammatory biomarkers after 5 h of exposure to PM_2.5–10_, PM_10_ endotoxins, gaseous pollutants (O_3_ and NO_2_), elemental and organic carbon, oxidative potential and trace and inorganic secondary metals. After two and 18 h of exposure they observed an association between of IL-6 and IL-8 in NL with, PM_10_ endotoxins, organic carbon and nitrate.

Another finding of this study was the direct association between eosinophil and rhinitis symptoms in 3 months of HP. It is suggested that eosinophils are predominant in this type of inflammation, especially in the late phase of nasal response to occupational allergens.^20^, ^41^ These are known as primary effector cells in the pathogenesis of allergic rhinitis and it is considered that the process of recruitment into the nasal epithelium and sub mucosa combined results in several signaling molecules and cells.

The study of Castano et al.^20^ investigated the type and kinetics of the nasal inflammatory response after exposure to occupational allergens and a significant increase in the percentage of eosinophil at 30 min after exposure to occupational allergens was observed. No significant changes in nasal neutrophil levels were observed. Findings corroborate the study of Zuurbier et al.^42^ that evaluated 34 healthy individuals exposed to two hours of contact to vehicular traffic, where there was an inverse association between number of particles and PM_2.5_ with neutrophils. This may explain, at least in part, the findings of our study, which documented the direct association with eosinophil and inverse with neutrophils in three months of HP.

Therefore, this study hopes to contribute and encourage preventive and interventionist procedures in the health of this population, especially on the risks of rhinitis symptoms, which may precede the development of occupational asthma. As a limitation of the study, it is possible to indicate the lack of the use of a questionnaire validated in the Portuguese language for symptoms of rhinitis in adults.

## Conclusion

In summary, the results showed that during three months of working in the burnt sugarcane harvesting there was an increase on the prevalence of rhinitis symptoms and higher concentrations of IL-6 at the local level; furthermore, eosinophil counts were directly associated with the rhinitis symptoms in the period of higher concentration of particulate matter.

## Funding

This work was supported by Fundação de Amparo à Pesquisa do Estado de São Paulo (FAPESP; grant: 2014/08029-0).

## Conflicts of interest

The authors declare no conflicts of interest.
